# Inheritance and fitness costs of resistance to *Bacillus thuringiensis* toxin Cry2Ad in laboratory strains of the diamondback moth, *Plutella xylostella* (L.)

**DOI:** 10.1038/s41598-019-42559-2

**Published:** 2019-04-16

**Authors:** Jinying Liao, Yiqun Xue, Guangjing Xiao, Miao Xie, Shuting Huang, Shijun You, Kris A. G. Wyckhuys, Minsheng You

**Affiliations:** 1 State Key laboratory of Ecological Pest Control for Fujian and Taiwan Crops, Institute of Applied Ecology, Fujian Agriculture and Forestry university, Fuzhou, 350002 China; 2 Joint International Research Laboratory of Ecological Pest Control, Ministry of Education, Fuzhou, 350002 China; 3 Key Laboratory of Green Pest Control (Fujian Agriculture and Forestry University), Fujian Province University, Fuzhou, 350002 China

## Abstract

The diamondback moth, *Plutella xylostella* (Lepidoptera: Plutellidae), is one of the main pests of *Brassica* crops worldwide. Management of *P. xylostella* is particularly challenging, as different field populations have readily acquired resistance to a wide range of insecticides, including *Bacillus thuringiensis* (Bt) toxins. In this study, a novel strain of *P. xyllostela* (Fuzhou-R2Ad) with 120-fold resistance to Bt Cry2Ad was selected in the laboratory, after screening for 66 generations from the susceptible strain Fuzhou-S. In the absence of Bt Cry2Ad toxin, the Fuzhou-R2Ad had significantly lower fitness as compared to the susceptible strain, which might be related to induced genetic changes to Bt toxins. We used several models to measure the dominance levels of insecticide resistance among different strains and found an incompletely recessive inheritance pattern of the Fuzhou-R2Ad resistance, which might be controlled by multiple genes. This study constitutes the first report of laboratory-acquired resistance to Cry2Ad toxin in *P. xylostella*. Our work presents further insights into the mechanism of Bt resistance and has immediate implications for the integrated pest management of *P. xylostella* globally.

## Introduction

The diamondback moth (DBM), *Plutella xylostella* (L.) (Lepidoptera: Plutellidae), is one of the world’s most destructive pests of *Brassica* crops, and causes an estimated cost of US$4-5 billion annually in direct damage and pest management globally^1,2^. Although there are multiple tactics for DBM management, chemically-synthesized insecticides remain the most common and widely-used approach. As insecticide-based management has caused substantial resistance problems in DBM^3–7^, biological pesticides are increasingly promoted as sustainable and environmentally-friendly alternatives. More specifically, the use of *Bacillus thuringiensis*, a soil-dwelling bacterium, offers durable and effective pest control without negative side effects on humans, vertebrates and most beneficial organisms^8,9^. This also has led to the development of genetically-modified (GM) crops, using Bt genes that biosynthesize the toxic crystalline (Cry) protein. However, given the ability of DBM to rapidly develop resistance to insecticides, there is significant concern that this pest could equally inherit and sustain resistance to Bt toxins.

A lot of research has been conducted on the genetic basis of insect resistance to Bt toxins^10–14^. The work has shown that a high level of resistance is primarily conferred through one or several autosomal genes, which are either recessive or incompletely recessive^6,15,16^. In contrast, the relatively low resistance is acquired through dominant inheritance mechanisms^17,18^. Four different models have been defined for insecticide resistance and dominance, based on phenotypic traits. First, a *D*_LC_ model was applied for insecticide resistance, centred on LC_50_ values of dose-mortality curves^19–21^. Next, Roush and McKenzie developed an effective dominance *D*_ML_ model by assessing mortality at a particular dose of a given insecticide^22^. Third, the relative fitness of dominance *D*_WT_ was calculated based upon the fitness of particular genotypes in insecticide-treated areas^23,24^. Last, a general formula has been proposed for dominance levels in relation to insecticide resistance^25^. Overall, dominance levels can be calculated for different traits, including insect fitness in insecticide-treated or untreated area *D*_WNT_. Although the dominance level could be estimated by *D*_LC_, *D*_ML_, *D*_WT_ and *D*_WNT_, it may still be varied by environmental influences, genetic information and the selection of an insecticide resistance allele. Using *D*_LC_ and *D*_ML_ models, it has been shown that the resistance in Cry1Ac-selected strains was incompletely recessive in a field-derived population of DBM^6^. Pereira *et al*. has demonstrated a recessive inheritance of Cry1F resistance in European corn borer *Ostrinia nubilalis*, which was indicated by a dominance level *D*_LC_ less than 0.11^16^. However, to our knowledge, there are no published studies that utilize the various models, especially *D*_WNT_, to fully evaluate the degree of the dominance.

In the present study, we evaluate, for the first time, the inheritance properties of a laboratory DBM strain with high resistance to Bt Cry2Ad, by comparing dominance of insecticide resistance between the susceptible strain, the positive and negative cross of the resistant strain and the backcross. Furthermore, we investigate levels of dominance and inheritance of resistance to Cry2Ad toxin in the hybrid, resistant and susceptible strains without selection pressure. Additionally, we estimate whether inheritance of Bt Cry2Ad resistance in *P. xylostella* is controlled by a single-gene or multiple genes. The results of this research have direct implication for resistance management of DBM to Cry2Ad, and can provide further information to advance the effective control of DBM globally.

## Materials and Methods

### Cry Toxin

Cry2Ad toxin was obtained from a Bt strain, BRC-HZP10, which was supplied by the Key Laboratory of Biopesticide and Chemical Biology, Fujian Agriculture and Forestry University (Fuzhou, China). The purity of the extracted Cry2Ad protein reached 88.34%^26^. Prior to its use in the experiments, Cry2Ad toxin was prepared in 0.2% Triton X-100.

### Insect strains

A susceptible strain of *P. xylostella*, Fuzhou-S, was collected in 2004 from fields of cabbage (*Brassica oleracea* var. capitata) in Fuzhou (Fujian, China; 26.08°N, 119.28°E). Whole-genome sequencing was applied to characterize the full genomic mapping^27^. The Fuzhou-S strain has been kept for over 150 generations under greenhouse conditions without exposure to insecticides, with individuals reared on potted radish seedlings (*Raphanus sativus* L. var. sativus) under the condition of 25° ± 1 °C, 65 ± 5% RH and 16 L:8D photoperiod.

A resistant strain was derived from the Fuzhou-S strain, by exposing the 3rd instar larvae of DBM to *R. sativus* leaves treated with Cry2Ad toxin. Fresh and untreated *R. sativus* leaves were dipped into the Cry2Ad toxin protein solution at LC_75_ concentration for 10 s, and excess solution was wiped off with filter paper. After 48 h, the surviving larvae were then selected, allowed to pupate and chosen for production of further progeny^28^. Similar to the Fuzhou-S strain, the resistant Fuzhou-R2Ad strain has been maintained for about 70 generations in the laboratory without any exposure to insecticides except for Cry2Ad.

### Bioassay

Following the procedures as outlined above, *R. sativus* leaves (ca. 10 mm diameter) were treated with five gradient concentrations of Cry2Ad solution. After drying, leaves were fed to the 3^rd^-instar *P. xylostella* larvae that had previously been starved in clear plastic cups (78 mm (top) and 51 mm (bottom) in diameter, 82 mm height) for 2 h^29–31^. Each concentration was tested for a batch of 12 DBM larvae, and the experiments were independently repeated three times with 10 leaves in each replicate. In a control group, larvae were fed with leaf disks (ca. 10 mm diameter) that had been treated with distilled water containing 0.2% Triton X-100.

The treated larvae were then transferred to a climate chamber at 25° ± 1 °C, 65 ± 5% RH, and a 16 L:8D cycle. After 48 h, fresh untreated *R. sativus* leaves were added. Mortality of larvae was recorded after 72 h, and a toxicity regression curve was developed to estimate the value of LC_50_ with 95% confidence intervals.

### Hybridization

After pupation, each pupa was transferred individually into a collection tube for further eclosion. Emerged adults were sexed, and used for production of a F1 generation through reciprocal mass crosses. For one cross, 30 Fuzhou-R2Ad females were allowed to mate with 30 Fuzhou-S males in one laying cage (100 mm diameter and 80 mm height). For a second cross, 30 Fuzhou-S females were paired with 30 Fuzhou-R2Ad males^32^, larvae from the two parental colonies were defined as F1 (Fuzhou-R2Ad♀ × Fuzhou-S♂) and F1’ (Fuzhou-R2Ad♂ × Fuzhou-S♀), and subject to the above bioassays. Subsequently, F2 progeny was obtained through single-pair crosses between F1 progeny, and a backcross (BC) was produced by pairing a F1 hybrid with the Fuzhou-S strain (F1 × Fuzhou-S). Lastly, 20 susceptible adults (i.e., 10 females and 10 males) were mixed with 20 resistant adults (10:10 sex ratio) for a pooled hybrid (R × S). Dominance of Cry2Ad toxin resistance in F1, F1’ and BC hybrids were determined based on the probit analysis (visualised by slopes of log dose–probit line (LD-P line)), LC_50_ value and corresponding 95% confidence limits.

### Fitness tests

Newly-hatched larvae from Fuzhou-S, Fuzhou-R2Ad, F1 and F1’ hybrid populations were randomly chosen, and individualized on potted turnip sprouts (ca. 40 mm diameter). On a daily basis, development of *P. xylostella* was monitored and the relevant biological parameters, including mortality, pupation rate, eclosion rate, and adult sex ratio, were recorded. Single-pair crosses of *P. xylostella* adults were conducted in 60 mm Petri dishes lined with moist filter paper, and mated females were allowed to lay eggs on the moist filter paper. Mated females were fed with 10% honey solution, and fecundity of each strain was recorded until all moths died.

Eggs were individually collected and incubated in Petri dishes, and egg eclosion rates were computed. Net population growth rate (*R*_0_) was determined, defined as the ratio of new larvae (*N*_n+1_) to the initial number (*N*_n_). The relative fitness of the resistant strain was calculated by:

$${\rm{Relativefitness}}={R}_{0}(\mathrm{resistant\; or\; hybrid\; strain})/{R}_{0}({\rm{susceptiblestrain}})$$

### Data analysis

For each bioassay, LD-P line, LC_50_ value, 95% confidence limits and the relative standard deviation were assessed. Two LC_50_ values are considered to be significantly different (*P* < 0.05) if their 95% confidence intervals do not overlap^33^.

Based on the LC_50_, the resistance ratio was defined as the ratio between the LC_50_ value of Fuzhou-R2Ad, F1 or BC and that of the susceptible strain (i.e., Fuzhou-S). Degree of dominance (*D*) at LC_50_ was calculated by:

$$D=(2{{\rm{logLC}}}_{{\rm{RS}}}-{{\rm{logLC}}}_{{\rm{R}}}-{{\rm{logLC}}}_{{\rm{S}}})/({{\rm{logLC}}}_{{\rm{R}}}-{{\rm{logLC}}}_{{\rm{S}}})$$

where LC_R_, LC_RS_ and LC_S_ represent lethal concentrations for resistant homozygotes, heterozygotes, and susceptible homozygotes, respectively. The value of *D* ranges from −1 to 1, representing a complete recessive towards an absolute dominance. Furthermore, *D*_LC_, was calculated by:

$${D}_{{\rm{LC}}}=({{\rm{logLC}}}_{{\rm{RS}}}-{{\rm{logLC}}}_{{\rm{S}}})/({{\rm{logLC}}}_{{\rm{R}}}-{{\rm{logLC}}}_{{\rm{S}}})$$

which is equal to (*D* + 1)/2^34^. Hence, the *D*_LC_ value varies between 0 (recessive resistance) and 1 (dominant resistance).

We equally applied the *D*_WT_ model to evaluate relative fitness of dominance under Bt insecticide selection. *D*_WT_ was calculated by:

$${D}_{{\rm{W}}{\rm{T}}}=({W}_{{\rm{T}}{\rm{R}}{\rm{S}}}-{W}_{{\rm{T}}{\rm{S}}{\rm{S}}})/({W}_{{\rm{T}}{\rm{R}}{\rm{R}}}-{W}_{{\rm{T}}{\rm{S}}{\rm{S}}})$$

where *W*_TSS_, *W*_TRS_ and *W*_TRR_ represent the relative fitness at a specific insecticide concentration for susceptible homozygotes, heterozygotes, and resistant homozygotes, respectively. If susceptible and resistant strains are considered as homozygous genotypes, *D*_WT_ will be taken as *h*^23,35^. In a similar fashion as *D*_LC_, the *h* value ranges from 0 to 1 (i.e., from completely recessive to completely dominant resistance).

Another approach was used to assess dominance. For instance, *D*_WNT_ value was calculated by:

$${D}_{{\rm{W}}{\rm{N}}{\rm{T}}}=({W}_{{\rm{N}}{\rm{T}}{\rm{R}}{\rm{S}}}-{W}_{{\rm{N}}{\rm{T}}{\rm{S}}{\rm{S}}})/({W}_{{\rm{N}}{\rm{T}}{\rm{R}}{\rm{R}}}-{W}_{{\rm{N}}{\rm{T}}{\rm{S}}{\rm{S}}})$$

where *W*_NTSS_, *W*_NTRS_ and *W*_NTRR_ represent relative fitness in the absence of insecticide for susceptible homozygotes, heterozygotes, and resistant homozygotes, respectively^25^. When the D_*WNT*_ value is 0.5, resistance is called co-dominant. *D*_WNT_ values ranging from 0 to 0.5 demonstrate partial recessive, while *D*_WNT_ values between 0.5 to 1 refer to partial dominance.

To test the genetic mode of inheritance, the expected mortality (*E*) of BC and F2 under a certain concentration of insecticide was estimated according to Georghiou’s method^36^.

$${E}_{{\rm{BC}}}=({W}_{1}+{W}_{2})\times 0.5$$

$${E}_{{\rm{F2}}}=({W}_{1}+{W}_{2}+{W}_{3})\times 0.25$$

in which *W*_1_, *W*_2_, *W*_3_ represent the actual mortality of Fuzhou-S, Fuzhou-R2Ad, and F1, respectively, for a given dose of insecticide. Chi-square test was employed to compare observed and expected mortality of BC and F2^37^. All of the above analyses, including one-way ANOVA with post-hoc Tukey’s honestly significant difference, were performed by using data processing system (DPS) V9.01, while figures were developed using Prism Graphpad 6.

## Results

### Cry2Ad resistance ratio

The resistance to Cry2Ad developed slow, and increased 1.04 times at the 12^th^ generation as compared to the susceptible strain (Table 1). Resistance gradually increased over subsequent generations and by generation 37 a 8.70-fold increase was observed over the susceptible strain. In the 66^th^ generation, the relative resistance ratio was 120.59 (Table 1).

**Table 1:** Resistance ratio of *P. xylostella* to Cry2Ad over multiple generation selection as compared to the susceptible Fuzhou-S strain.

Generation	number of insects tested	Slope ± SE	LC_50_ (95% fiducial limits) (ng/mL)	RR*	*P* (df = 3)
0	216	4.34 ± 0.50	6.65(5.58–8.28)	1.00	0.8812
12	216	1.78 ± 0.26	6.92(4.83–9.11)	1.04	0.9964
16	216	4.54 ± 0.38	32.35(26.43–37.92)	4.86	0.6909
27	216	1.68 ± 0.27	51.53(32.94–70.37)	7.76	0.9999
37	216	2.35 ± 0.32	57.79(41.96–73.00)	8.70	0.9998
41	216	2.13 ± 0.28	120.20(96.79–157.82)	18.10	0.9973
52	216	2.35 ± 0.32	154.45(123.84–200.47)	23.26	0.9058
66	216	1.26 ± 0.31	800.73(372.94–6142.62)	120.59	0.9633

*RR (resistance ratio) is calculated as LC_50_ (Fuzhou-R2Ad, F1 or BC)/LC_50_ (Fuzhou-S). LC_50_(Fuzhou-S) is expressed as 6.65 ng/mL. Each LC_50_ value represents the average of 8 independent measurements.

### Biological fitness parameters

In the Fuzhou-S strain, survival rates (% ± standard error) of the 1^st^, 2^nd^, 3^rd^ and 4^th^ instar larvae were 90.30 ± 0.50, 57.80 ± 0.77, 93.23 ± 1.63, and 91.00 ± 1.59, respectively (Table 2). For the resistant Fuzhou-R2Ad strain, corresponding survival rates (%) were 67.25 ± 0.59, 58.71 ± 0.19, 100.00 ± 0.12 and 74.90 ± 1.97, respectively. Survival rates of 1^st^ and 4^th^ instar larvae of the Fuzhou-R2Ad strain were significantly lower than those of the Fuzhou-S strain, and the relative fitness (*D*_WT_) of the Fuzhou-R2Ad strain was 0.29.

**Table 2:** Population growth parameters of different *P. xylostella* strains.

Biological characteristics	Fuzhou-S	Fuzhou-R2Ad	F1	F1'	F value	*P*
Initial amount of eggs	140	186	118	87		
Egg hatch (%)	80.72 ± 1.22^bB^	87.08 ± 0.36^aA^	82.26 ± 0.57^bAB^	74.57 ± 0.97^cC^	36.82	0.0001
Survival rate 1st instar (%)	90.30 ± 0.5^aA^	67.25 ± 0.59^cC^	82.82 ± 0.89^bB^	85.01 ± 1.27^bAB^	127.19	0.0001
Survival rate 2nd instar (%)	57.80 ± 0.77^cC^	58.71 ± 0.19^cC^	97.57 ± 1.22^aA^	85.74 ± 1.01^bB^	498.22	0.0001
Survival rate 3rd instar (%)	93.23 ± 1.63^bB^	100.00 ± 0.12^aA^	96.30 ± 0.15^abAB^	100.00 ± 1.03^aA^	15.99	0.0010
Survival rate 4th instar (%)	91.00 ± 1.59^aA^	74.90 ± 1.97^cC^	84.22 ± 0.54^bAB^	76.59 ± 1.05^cBC^	28.22	0.0001
Number of pupae	17.00 ± 0.67^abAB^	16.00 ± 1.00^abAB^	21.00 ± 1.00^aA^	12.00 ± 2.08^bB^	8.02	0.0085
Pupation rate (%)	33.42 ± 1.62^cC^	25.77 ± 1.02^cC^	53.71 ± 1.47^bB^	83.98 ± 2.46^aA^	227.33	0.0001
Adult number	14 ± 0.67^abAB^	12 ± 0.58^bAB^	18.00 ± 1.00^aA^	10 ± 1.53^bB^	11.49	0.0029
Emergence rate (%)	86.11 ± 3.87^aA^	75.18 ± 2.43^aA^	86.42 ± 0.72^aA^	83.99 ± 2.46^aA^	4.06	0.0502
Sexual ratio (female:male)	1.17^aA^	1.00^aA^	1.31^aA^	1.50^aA^		
Fecundity/female	102 ± 3.67^abAB^	91 ± 5.29^bAB^	129 ± 7.21^aA^	82 ± 8.97^bB^	9.65	0.0049
Number of offspring eggs	1414	546	1062	574	—	—
R0	10.10	2.93	9.00	6.60	—	—
Relative fitness	1.00*	0.29	0.89	0.65		

According to one-way with post-hoc Tukey’s honestly significant difference, the same superscript letter following the numbers between rows of a given column indicates no significant difference between the strains at *P* > 0.05. The different upper and lower case letters stand for the significance with *P* < 0.01, and *P* < 0.05, respectively.

*Relative fitness of the susceptible Fuzhou-S strain is defined as 1.

Other fitness parameters, such as egg hatch rate, survival rate of the 2^nd^-instar larvae, pupation rate, and female fecundity were significantly higher in F1 hybrid compared to F1’. And the relative fitness values of the positive cross F1 and negative cross F1’ were 0.89 and 0.65, respectively.

### Inheritance properties

All experimental strains proved susceptible to Cry2Ad, and no significant difference was recorded in LC_50_ values between F1 and F1’ strains (Table 3). In the pooled hybrid (R × S), the LC_50_ value was significantly lower than that of Fuzhou-R2Ad strain. Also, the overlap in 95% confidence limits of LC_50_ between F1 and F1’ strains confirmed that Cry2Ad resistance was autosomally inherited, without maternal effects and sex linkage.

**Table 3:** Susceptibility to Cry2Ad toxin in a susceptible strain (Fuzhou-S), resistant strain (Fuzhou-R2Ad), and different reciprocal crosses of the *P. xylostella* strains.

Strain or cross	Number of insects tested	Slope ± SE	LC_50_ (95% fiducial limits) (ng/mL)	RR*	*P* (df = 3)
Fuzhou-S	216	1.44 ± 0.25	9.84 (6.98–13.61)	1.00	0.8874
Fuzhou-R2Ad	216	1.26 ± 0.31	800.73 (372.94–6142.62)	81.37	0.9633
F1 (Fuzhou-R2Ad♀ × Fuzhou-S♂)	216	1.39 ± 0.22	230.27 (155.81–457.35)	23.40	0.9737
F1’ (Fuzhou-R2Ad♂ × Fuzhou-S♀)	216	1.15 ± 0.25	116.91 (77.44–187.60)	11.88	0.8206
R × S (pooled)	432	1.27 ± 0.23	173.59 (116.62–322.47)	17.64	—
S × F1 (F1♀ × S♂)	216	0.83 ± 0.24	297.84 (160.45–1591.57)	30.27	0.9696
F2 (F1 × F1)	216	1.14 ± 0.25	77.71 (53.83–107.38)	7.90	0.8943

Resistance ratio is presented by LC50 of a given strain or cross divided by LC50 of the susceptible Fuzhou-S strain.

### Estimation of dominance

Upon testing five different Cry2Ad toxin concentrations, LC_50_ values for F1 and F1’ progenies yielded *D*_F1_ = −0.73, *D*_F1′_ = −0.44, *D*_LC-F1_ = 0.13, *D*_LC-F1′_ = 0.28. The effective dominance (*h*) varied between 0.33 up to 0.71, and negatively correlated with the Cry2Ad protein concentration (Table 4). Based on the relative DBM fitness (Table 2), the respective fitness values of F1 and F1’ in insecticide-treated areas *D*_WNT_ were 0.15 and 0.49. Hence, *D*, *D*_LC_, and *D*_WNT_ parameters indicate that the genes conferring resistance to Cry2Ad in DBM selected strain was incompletely recessive. However, when subject to Cry2Ad at 25.32-202.60 μg/ml, DBM larvae had relatively high *h* values (0.56–0.71), suggesting an incomplete dominant inheritance of the Cry2Ad resistance.

**Table 4:** Effective dominance (*h*) of resistance to Cry2Ad in different strains of *P. xylostella*, as compared to Fuzhou-R2Ad.

Concentration of Cry2Ad (ng/ml)	Strain or cross	Survival (%)	Fitness	*h*
25.32	Fuzhou-S	27.80 ± 1.66	0.28	
	Fuzhou-R2Ad	97.22 ± 1.60	1.00	
	F1(Fuzhou-R2Ad♀ × Fuzhou-S♂)	77.14 ± 1.19	0.79	0.71
50.65	Fuzhou-S	15.30 ± 1.48	0.16	
	Fuzhou-R2Ad	94.44 ± 0.00	1.00	
	F1(Fuzhou-R2Ad♀ × Fuzhou-S♂)	65.71 ± 2.83	0.69	0.64
101.30	Fuzhou-S	7.30 ± 1.42	0.08	
	Fuzhou-R2Ad	86.11 ± 2.78	1.00	
	F1(Fuzhou-R2Ad♀ × Fuzhou-S♂)	51.43 ± 2.89	0.60	0.56
202.60	Fuzhou-S	3.00 ± 1.52	0.04	
	Fuzhou-R2Ad	75.00 ± 2.78	1.00	
	F1(Fuzhou-R2Ad♀ × Fuzhou-S♂)	45.71 ± 2.03	0.61	0.59
405.21	Fuzhou-S	1.01 ± 0.71	0.01	
	Fuzhou-R2Ad	66.67 ± 3.21	1.00	
	F1(Fuzhou-R2Ad♀ × Fuzhou-S♂)	22.86 ± 1.28	0.34	0.33

Mortality (%) is calibrated before fitness calculation, and it is calculated as (*W*_RR_ − *W*_RS_)/(*W*_RR_ − *W*_SS_), where *W*_RR_, *W*_RS_, and *W*_SS_ represent fitness values at a specific toxin concentration.

### Genetic mode of inheritance

**Figure 1:**
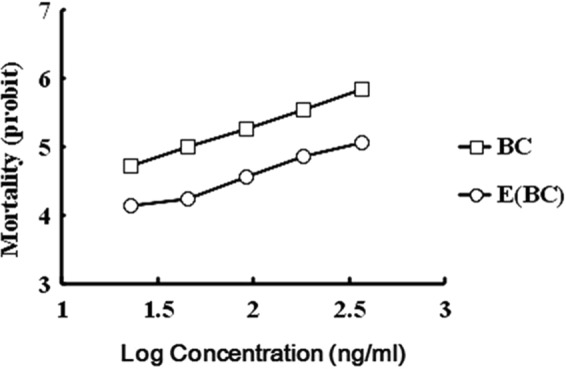
The slopes of log dose–probit lines (LD-P lines) for BC and the expected LD-P line of BC progeny (E_BC_). Expected mortality at concentration x ng/ml is calculated as 0.5 × (mortality of F1 at x ng/ml + mortality of Fuzhou-S at x ng/ml), obtained from regression lines of parental strains.

**Figure 2:**
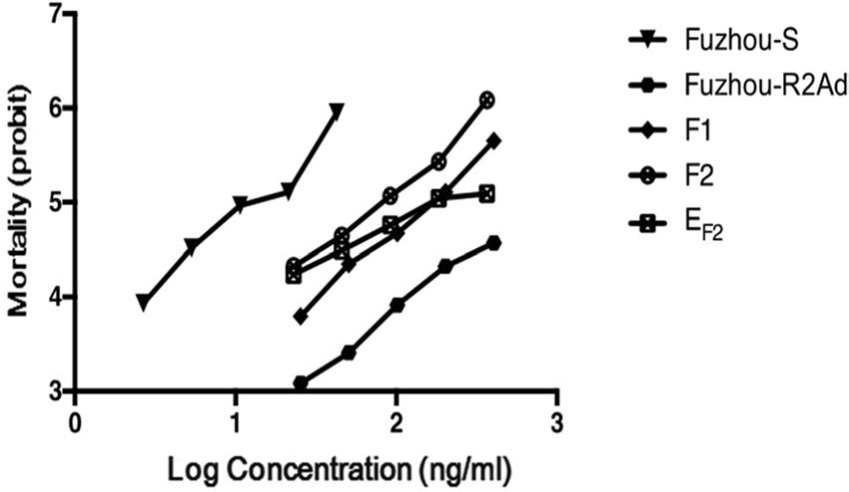
LD-P lines for susceptible (Fuzhou-S) and resistant parents (Fuzhou-R2Ad), F1, F2 and expected LD-P line of F2 progeny. Expected mortality at concentration x ng/ml is calculated as 0.25 × (Fuzhou-S mortality + Fuzhou-R2Ad mortality + F1 mortality), obtained from regression lines of parental strains.

LD-P lines and expected values were distinguishable for both BC and F2 crosses (Figs 1 and 2). A plateau was not reached neither after the 50% mortality of BC progeny nor at 25% or 75% mortality levels of F2 hybrids. Chi-square analysis showed that the resistance heredity in experimental DBM strains may be controlled by multiple genes (Tables 5 and 6).

**Table 5:** Observed and expected mortality of the BC strain of *P. xylostella* treated with Cry2Ad, as evaluated with a Chi square test (χ^2^).

Concentration of Cry2Ad (ng/ml)	Observed		Expected		χ^2^	*P*
	Dead	Alive	Dead	Alive		
22.93	7	29	28	44	3.30	0.0692
45.86	8	28	36	36	6.56	0.0104
91.72	12	24	43	29	5.67	0.0172
183.44	16	20	51	21	6.02	0.0141
366.88	19	17	58	14	7.74	0.0054
∑χ^2^					29.29	

The single gene conferring Cry2Ad resistance is defined as the Chi-square hypothesis.

**Table 6:** Observed and expected mortality of F2 strain of *P. xylostella* treated with Cry2Ad.

Concentration of Cry2Ad (ng/ml)	Observed		Expected		χ^2^	*P*
	Dead	Alive	Dead	Alive		
22.93	9	27	16	56	0.01	0.9357
45.86	13	23	22	50	0.13	0.5663
91.72	19	17	29	43	1.05	0.2222
183.44	24	12	37	35	1.70	0.1923
366.88	31	5	46	26	4.76	0.0131
∑χ^2^					7.65	

## Discussion

A thorough understanding of pesticide resistance development in *P. xylostella* is crucial for an effective and sustainable management of this globally-important pest. Past research has shown that the development of Bt resistance depends on the particular Bt strain and the type of Bt toxin^38^. Induced by Bt subspecies *kurstaki*, the resistance ratio of *P. xylostella* strain NO was 30 times^3^. Another *P. xylostella* strain NO-95 selected with high resistance to Bt subspecies *kurstaki* has very low resistance to Bt subspecies *aizawai*^5^. In 2014, a Cry1Ie susceptible *Ostrinia furnacalis* strain of ACB-BtS was found to have cross resistance to Cry1Ab, Cry1Ac and Cry1F toxins^39^. Other work has shown that a given Bt toxin produced by the same Bt species may exhibit different impacts on a DBM strains/populations, due to the differential modes of action of the Bt toxins^40,41^. In this study, we determine that DBM resistance development to the Bt Cry2Ad toxin is possible, after laboratory-based screening for 5 years and 66 generations. The resulting Fuzhou-R2Ad resistant strain had 120.59 times higher levels of resistance than the susceptible Fuzhou-S strain.

When unexposed to Bt Cry2Ad toxin, the Fuzhou-R2Ad has significantly lower fitness as compared to the susceptible strain. Similar findings has been made with DBM populations in Hawaii, where Dipel 2X® (a wettable powder formulation of *B. thuringiensis* subsp. *kurstaki* strain HD-l) resistant strain NO-QA exhibited reduced survival, egg hatching and mating rates^42^. Such reduction in fitness is possibly related to induced genetic changes to Bt toxins, which may remain even in the absence of selection pressure^24,43^. Hence, it is possible that effective DBM pest control can still be attained for resistant populations by discontinuing Bt Cry2Ad applications.

Inheritance of Bt resistance in the diamondback moth is considered to occur autosomally^14,28,44^, and similar inheritance models have been recorded for the Asian corn borer *Ostrinia furnacalis*^38^, the southern house mosquito *Culex quinquefasciatus*^45^, and the cotton bollworm *Helicoverpa armigera*^46,47^. As one notable exception, Malaysian populations of *P. xylostella* exhibited maternal effects on Cry1Ac resistance development^3^. In the current research, we detect susceptibility to Cry2Ad in all experimental strains or crosses, and confirm this to be autosomal resistance to Cry2Ad, without maternal effects or sex linkage (Table 3).

Our work also show that the resistance inheritance to Cry2Ad toxin in DBM strains is incompletely recessive. This is clearly shown by the following parameters: *D*_F1_ values of 0.73 and 0.13, *D*_LC_ values of 0.44 and 0.28, *D*_WNT_ values of 0.15 and 0.49 for F1 and F1’ respectively. *D*, *D*_LC_ and *D*_WNT_ values indicate that resistance to Cry2Ad in the Fuzhou strains of *P. xylostella* is partially recessive. Secondly, the effective dominance is negatively regulated by concentrations of the Bt toxin^48,49^, namely an incomplete recessivity of resistance at a high Cry2Ad level and an incomplete dominance at low concentrations of Cry2Ad protein. However, when DBM populations are treated with a low dose of toxin, the reduced selection pressure may cause bias because of the increased survival rate in the susceptible strain.

Our work constitutes the first report of Cry2Ad resistance in *P. xylostella*, sheds light upon Bt resistance development, and could guide further pest management interventions against a globally-relevant lepidopteran pest. Caution needs to be taken when extrapolating our findings, as our research is conducted under highly-artificial conditions with laboratory-reared individuals. Hence, one could still encounter an incompletely coincident resistance to Cry2Ad due to variations in DBM field populations^50^. Further, we postulate that resistant heredity in local diamondback moth populations is conferred by multiple genes (Figs 1 and 2; Table 5). All of the above provide fundamental insights into the mechanism and evolution of Bt resistance, according to the neo-Darwinian theory^51^. Further investigation of Bt resistance genes through molecular biology approaches, including molecular marker selection, would be a great help for the genetic manipulation of the diamondback moth. Moreover, the knowledge obtained from this research could boost the effectiveness of pest management interventions and enable sustainable DBM control globally.
